# Active infective endocarditis of a bicuspid aortic valve causing left ventricular outflow tract pseudoaneurysm and right atrium shunt: A case report

**DOI:** 10.1016/j.ijscr.2021.106527

**Published:** 2021-10-18

**Authors:** Tsubasa Mikami, Daisuke Yoshioka, Takuji Kawamura, Koichi Toda, Yoshiki Sawa, Shigeru Miyagawa

**Affiliations:** Department of Cardiovascular Surgery, Osaka University Graduate School of Medicine, Suita, Osaka, Japan

**Keywords:** LVOT, Left ventricular outflow tract, MAIVF, Mitral-aortic intervalvular fibrosa, Left ventricular outflow tract pseudoaneurysm, Infective endocarditis, Bicuspid aortic valve, Case report

## Abstract

**Introduction and importance:**

Left ventricular outflow tract pseudoaneurysm associated with infective endocarditis is a rare but life-threatening condition.

**Case presentation:**

A 68-year-old man developed infective endocarditis of a bicuspid aortic valve with suspected annulus abscess and was transferred to our department for further treatment. Cardiac workup revealed the formation of a left ventricular outflow tract pseudoaneurysm penetrating the right atrium. We successfully treated the patient with pseudoaneurysm repair using a bovine pericardium patch in combination with aortic valve replacement. The patient was uneventfully discharged after 6-week antibiotic therapy and remained well for the following 2 years.

**Clinical discussion:**

Surgery is the recommended treatment for left ventricular outflow tract pseudoaneurysms. Accurate diagnosis and identification of the anatomical conditions are crucial for determining the appropriate treatment.

**Conclusion:**

When considering the appropriate surgical treatment for left ventricular outflow tract pseudoaneurysm associated with infective endocarditis, pseudoaneurysm repair using a bovine pericardial patch and concomitant aortic valve replacement can be an effective and feasible therapeutic option.

## Highlights

• Infective endocarditis can cause left ventricular outflow tract pseudoaneurysms.
• Surgery is recommended for left ventricular outflow tract pseudoaneurysms.
• Accurate anatomical diagnosis is crucial for determining the appropriate treatment.

## 1 Introduction

Left ventricular outflow tract (LVOT) pseudoaneurysm is mostly caused by infective endocarditis, aortic valve or root surgery, and congenital heart disease [1]. It is a rare but life-threatening condition, and appropriate preoperative diagnosis and surgical intervention are crucial to improve the prognosis [1]. Herein, we report a case in which active infective endocarditis of a bicuspid aortic valve caused LVOT pseudoaneurysm and an intracardiac shunt to the right atrium, successfully treated with pseudoaneurysm repair using a bovine pericardium patch in combination with aortic valve replacement. This report has been reported in line with the SCARE criteria [2].

## 2 Presentation of case

A 68-year-old man, who developed septic arthritis of the knee, was admitted to a local hospital for antibiotic therapy and surgical debridement of the knee, suffering from refractory, persistent high fever without heart failure symptom for approximately 1 month. Transthoracic echocardiography revealed infective endocarditis of the aortic valve with a suspected annulus abscess. Therefore, the patient was transferred to our department for further treatment.

**Fig. 1:**
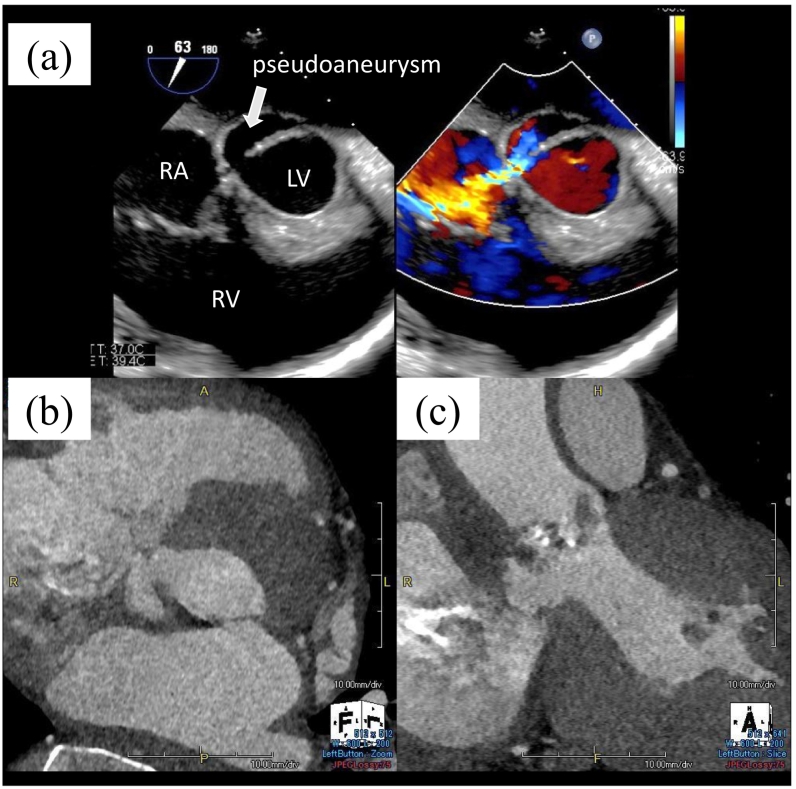
Preoperative transesophageal echocardiography and computed tomography images. An abnormal cavity around the aortic root leading to the left ventricle and right atrium, with the thickened wall (a) (LV: left ventricle, RA: right atrium, RV: right ventricle). The left ventricular outflow tract pseudoaneurysm penetrated the right atrium without pericardial effusion (b: axial, c: coronal).

Preoperative transthoracic echocardiography showed a bicuspid aortic valve with leaflet thickening and severe calcification, mild aortic regurgitation, and severe aortic stenosis with preserved left ventricular systolic function. In addition, an abnormal cavity around the aortic root leading to the left ventricle and right atrium was also detected. Transesophageal echocardiography revealed that systolic blood flow from the left ventricle flew into the right atrium thorough the cavity and that the wall of the cavity was thickened, although no obvious vegetation on the aortic and tricuspid valves was observed (Fig. 1a). Computed tomography confirmed the formation of an LVOT pseudoaneurysm penetrating the right atrium, but no pericardial effusion (Fig. 1b, c). Therefore, we diagnosed the patient as active infective endocarditis of the bicuspid aortic valve with suspected annulus abscess and a LVOT pseudoaneurysm penetrating the right atrium. The patient was transferred to the operating room for emergency surgery.

**Fig. 2:**
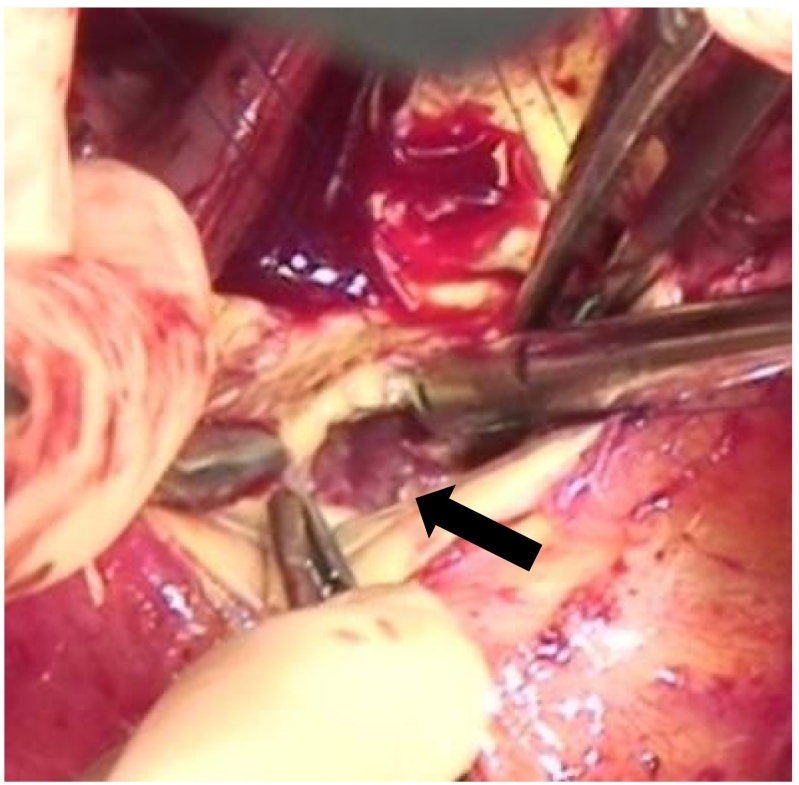
Intraoperative findings. The orifice of the left ventricular outflow tract pseudoaneurysm (black arrow) was relatively localised just below the non-coronary annulus.

**Fig. 3:**
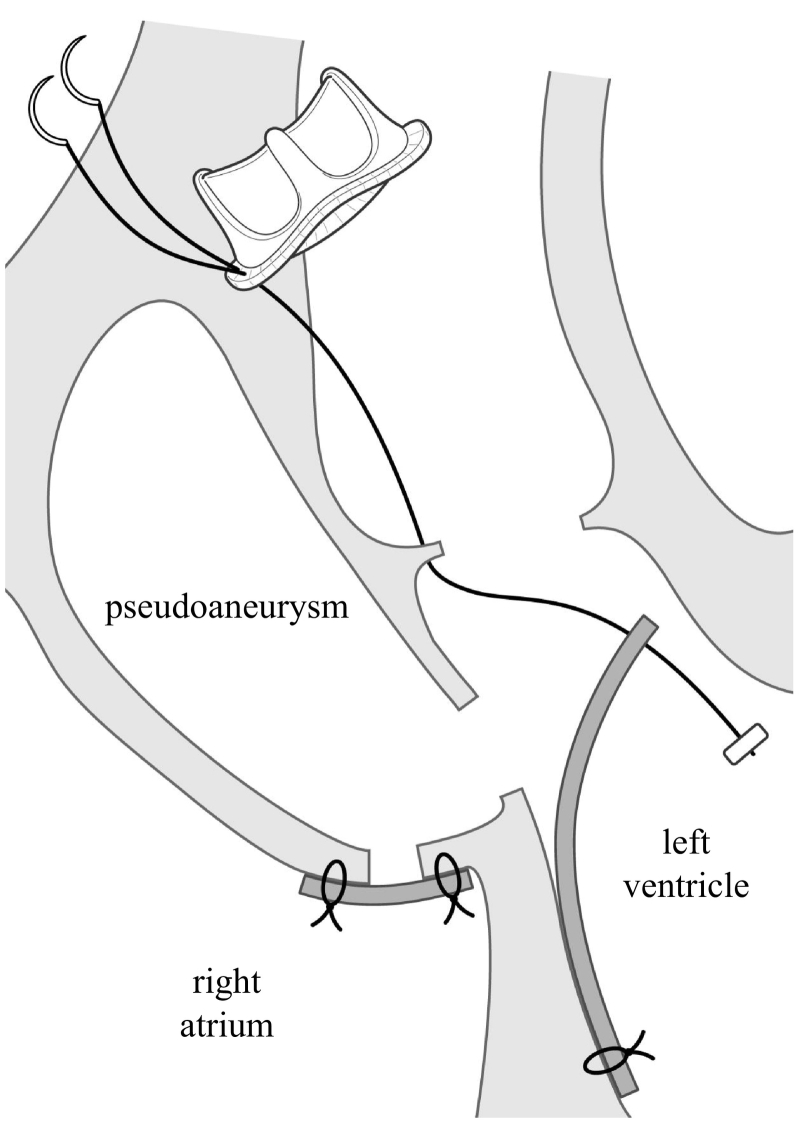
Schema of the surgical procedures. The anastomosis of the bovine pericardium patch to the lower edge of the pseudoaneurysm orifice and aortic annulus enabled us to reinforce the anastomosis of the prosthetic valve and exclude the pseudoaneurysm simultaneously.

During surgery, strong adhesions were found between the aortic root and the right atrium. After aortic cross-clamping and right atriotomy, we confirmed the leakage of antegrade cardioplegia administered to the ascending aorta from near the commissure of the anterior and septal tricuspid leaflet. It was also confirmed that the tricuspid valve was not infected. After cardiac arrest and aortotomy, we found a type 1 bicuspid aortic valve with fused right and left coronary cusps, severe calcification of the aortic valves and annulus, and infection of the right half of the non-coronary cusp, with attached vegetation. After meticulous removal of the aortic valves, we found the cavity penetrating the right atrium from beneath the aortic annulus and filled with infected granulation tissues (Fig. 2). We subsequently opened and thoroughly debrided the pseudoaneurysm from both sides of the left ventricle and right atrium. Thereafter, we anastomosed the tongue-shaped bovine pericardium patch to the mitral valve annulus and the left ventricular membranous portion located at the lower edge of the pseudoaneurysm orifice. Subsequently, aortic valve replacement was performed using a 23-mm INSPIRIS RESILIA aortic valve (Edwards Lifesciences, Irvine, CA, USA). In this procedure, we placed the upper edge of the bovine pericardium patch so that it was located just beneath the right half of the non-coronary annulus. By placing mattress sutures through the bovine pericardium patch and the aortic annulus to the Valsalva sinus, we reinforced the aortic annulus and the anastomosis of the prosthetic valve, and excluded the LVOT pseudoaneurysm simultaneously (Fig. 3). In addition, as the aortotomy was extended to just above the aortic annulus to secure a sufficient surgical field for intra-aortic procedures, we closed the aortotomy and repaired the fistula to the right atrium using a bovine pericardium patch.

Although the blood culture test performed before surgery was negative, the postoperative analysis of 16S ribosomal ribonucleic acid sequences for the intraoperatively resected aortic valve detected a bacterial gene suggestive of the *Streptococcus mitis* group. Postoperative transthoracic echocardiography showed favourable prosthetic valve function with no perivalvular leakage or residual shunt blood flow. The patient was uneventfully discharged without any complication, including conduction disorder, after 6-week antibiotic therapy and remained well for the following 2 years.

## 3 Discussion

LVOT pseudoaneurysm has been reported to be associated with infective endocarditis, aortic root or valve surgery, or congenital heart disease [1]. LVOT pseudoaneurysms typically occur in the mitral-aortic intervalvular fibrosa (MAIVF) [1], and most commonly lead to coronary artery compression and fistula formation in adjacent structures [3]. In the present case, the patient developed infective endocarditis related to the bicuspid aortic valve, and the infection spread over the aortic annulus to the interventricular septum side of the LVOT, resulting in the formation of an aortic root abscess and LVOT pseudoaneurysm penetrating the right atrium. LVOT pseudoaneurysms in the interventricular septum are rarer than those in the MAIVF [1], [4]. This may be due to a greater likelihood of the occurrence of an intracardiac shunt to the right atrium or ventricle, the so-called acquired Gerbode defect [5], than the formation of a pseudoaneurysm, when the interventricular septum is affected.

Surgery is the recommended treatment for LVOT pseudoaneurysms to prevent fatal complications [3]. The principles of surgical treatment are the elimination of the pseudoaneurysm orifice and replacement of the aortic valve, when involved [1]. In the present case, we performed the pseudoaneurysm repair with a bovine pericardial patch and concomitant aortic valve replacement due to the LVOT pseudoaneurysm orifice being relatively localised just below the non-coronary annulus and the involvement of severe aortic stenosis associated with the bicuspid aortic valve. Percutaneous transcatheter closure or transcatheter aortic valve replacement might have been an alternative therapeutic option if the infection was well-controlled and the patient had three aortic leaflets [6], [7], [8], [9], although it would be a very limited situation in reality.

## 4 Conclusion

We reported a case of active infective endocarditis of a bicuspid aortic valve causing an LVOT pseudoaneurysm and right atrium shunt that was successfully treated using a bovine pericardial patch and concomitant aortic valve replacement. When considering surgery for LVOT pseudoaneurysms associated with infective endocarditis in which the spread of infection under the aortic annulus is localised, repair using a bovine pericardial patch and concomitant aortic valve replacement can be an effective and feasible therapeutic option.

## Sources of funding

None.

## Ethical approval

This report was approved by the institutional review board of our hospital (approval no. 16105).

## Consent

Written informed consent was obtained from the patient for publication of this case report and accompanying images. A copy of the written consent is available for review by the Editor-in-Chief of this journal on request.

## Research registration

Not applicable. This case report is not a ‘First in Man’ study.

## Guarantor

Tsubasa Mikami, Daisuke Yoshioka, Shigeru Miyagawa.

## Provenance and peer review

Not commissioned, externally peer-reviewed.

## CRediT authorship contribution statement

Tsubasa Mikami drafted the manuscript. Daisuke Yoshioka revised the manuscript. Tsubasa Mikami, Daisuke Yoshioka (operator), and Koichi Toda (supervisor) performed the surgery and contributed to the perioperative care. All authors read and approved the final manuscript.

## Declaration of competing interest

None.
